# Effectiveness of Non-Pharmacological Interventions in Reducing Dental Anxiety Among Children with Special Needs: A Scoping Review with Conceptual Map

**DOI:** 10.3390/children12020165

**Published:** 2025-01-29

**Authors:** Zuhair Motlak Alkahtani

**Affiliations:** Department of Pediatric Dentistry and Orthodontic Sciences, College of Dentistry, King Khalid University, Abha 62529, Saudi Arabia; zmalqhtany@kku.edu.sa

**Keywords:** non-pharmacological interventions, special needs children, dental anxiety, behavior modification

## Abstract

Background: Children with special needs often need tailored approaches to oral healthcare to address their unique needs effectively. It is essential to analyze the effectiveness of non-pharmacological management in reducing dental anxiety among special needs children during dental treatment. Methods: Five electronic databases, PubMed, Scopus, Web of Science, Embase, and Google Scholar, were searched from 2007 to August 2024 for randomized control trials and observational studies comparing the effectiveness of non-pharmacological techniques in reducing dental anxiety during invasive and noninvasive dental treatment. The primary outcomes of the studied intervention were reduced dental anxiety and improved behavior during dental treatment. The conceptual map was created to understand the need for assessment and behavior management for special needs children (SN). Results: Nineteen articles qualified for the final analysis from 250 screened articles. Included studies evaluated the effect of strategies applied clinically, such as audio–visual distraction, sensory-adapted environment, and virtual reality. The included studies measured the trivial to large effect of measured interventions and supported non-pharmacological interventions in clinical settings. Conclusions: Most basic non-pharmacological interventions showed a trivial to large reduction in dental anxiety among SN patients. The conceptual map developed in this study supports the need for non-pharmacological interventions as they are cost-effective and create a positive environment in dental clinics. However, more studies need to focus on non-pharmacological behavior interventions in SN children to support the findings of this scoping review.

## 1. Introduction

Children with special needs (SN) are those who suffer from physical, mental, developmental, or behavioral conditions from birth and require constant medical care [1]. They often need tailored approaches to oral healthcare to address their unique needs effectively. Globally, around 20% of children have special healthcare needs, and around 65% of them face unmet dental needs compared to children without SN [2]. These disparities can be attributed to factors such as a lack of dental insurance, challenges with patient cooperation, and, most importantly, dentists’ perceptions regarding treatment provision [3]. Additionally, there is a scarcity of research focusing on the oral health conditions and management of children with special needs. Consequently, knowledge gaps exist concerning caries risk, dietary factors, oral hygiene practices, and appropriate management strategies. Therefore, guidelines are urgently needed to address the unmet dental needs and management of children with special needs.

Dental anxiety (DA) among children with special needs is considered one of the greatest challenges faced by general and pediatric dentists [4]. DA is a state of apprehension or fear that something unfavorable will occur during dental treatment. While dental anxiety is multifactorial and can affect anyone, it is especially prevalent among children dealing with congenital and developmental anomalies [5]. DA is reported as one of the major reasons for delaying dental treatment, which can lead to significant oral health issues, particularly in children with special needs. Moreover, children with special needs are more prone to dental anxiety, and dentists often employ non-pharmacological management techniques to address this challenge [2,5]. The document entitled “Behavior Guidance for the Pediatric Patients” states that SNCs require non-pharmacological guidance techniques that can help to reduce dental anxiety among this vulnerable population [6]. Prevalence rates for dental anxiety vary across populations but are generally reported between 5% and 15% in the general population [7]. However, for children with special needs, particularly those with autism spectrum disorder (ASD), developmental disabilities, and cognitive impairment, these rates are significantly higher due to sensory sensitivities, communication challenges, and difficulties in understanding or coping with dental procedures [6,7].

Globally, dentists actively try to incorporate non-pharmacological behavioral interventions to reduce dental anxiety among pediatric patients. The American Society of Pediatric Dentistry collectively termed these interventions as behavioral management techniques and divided them into two parts: 1. basic behavior guidance and 2. advance behavior guidance. Basic behavior guidance includes positive behavior toward dental treatment, tell–show–do techniques, non-verbal and verbal communications, ask–tell–ask, distraction, and desensitization [6]. Conversely, advanced behavior techniques include sedation, stabilization, and general anesthesia. Studies have reported various risk factors associated with advanced behavior techniques among children [3,7,8]. Due to the challenging behavior of children with special needs, it can be difficult for dentists to manage their dental care. In emergencies, dentists often resort to advanced behavior management techniques, which have certain drawbacks. As a result, parents typically prefer basic behavior guidance techniques for their children.

A systematic review of children with autism reported lacking evidence supporting basic behavioral techniques for their dental management [9]. Similarly, a systematic review of children with mild to moderate intellectual disabilities highlighted a lack of research on non-pharmacological management for this population [10]. A sequential systematic review reported that non-pharmacological interventions are effective when managing SNCs in dental settings. The authors of these reviews emphasized the need for guidelines to manage dental diseases among children with special needs effectively [11]. A pilot study on reduction in dental anxiety among children with the soap bubble technique found this distraction technique to be helpful [12].

Even though various studies focus on the oral health needs; SNCs, the development of non-pharmacological management in clinical settings is not much documented. Additionally, dental clinicians struggle to perform basic techniques such as dental diagnosis and prophylaxis without clear recommendations. The lack of effective non-pharmacological intervention increases the requirement for pharmacological management, leading to morbidity and decreasing dental access for this vulnerable group. Therefore, this scoping review aims to analyze the effectiveness of non-pharmacological management in reducing dental anxiety among special needs children.

## 2. Materials and Methods

The protocol for this scoping review was developed and submitted to an open science framework (https://doi.org/10.17605/OSF.IO/TMFX3, accessed on 18 December 2024). The research question developed for this scoping review was “What are the non-pharmacological interventions used to manage and reduce dental anxiety in children with special needs, and what is their reported effectiveness?” where the population of interest was children with special needs; the concept was non-pharmacological interventions to reduce dental anxiety, and the context was clinical settings.

A scoping review was chosen to answer this research question instead of a systematic review, as the primary aim was to identify gaps in the literature and develop a conceptual map to address the oral healthcare needs of SNC patients. Unlike systematic reviews, which focus on defining the evidence and measuring relationships between variables, scoping reviews are designed to explore the depth and breadth of research on complex topics and identify knowledge gaps. This scoping review followed the five-stage framework developed by Arksey and O’Malley (2019) [13] and adhered to the Preferred Reporting Items for Systematic Reviews and Meta-Analyses extension for Scoping Reviews (PRISMA-ScR) [14] (Supplementary Material Table S1).

### 2.1. Inclusion and Exclusion Criteria

Table 1 demonstrates the inclusion and exclusion criteria for the studies. Studies that showed comparisons between various non-pharmacological interventions were included in the current review. However, studies that compared pharmacological and non-pharmacological interventions were excluded. The focus of the current review was to assess the effectiveness of non-pharmacological interventions in reducing dental anxiety among SNCs.

### 2.2. Search Strategy

The developed PCC “population, context, and content framework” was used to analyze the initial search terms for various databases. A combination of MesH terminologies, keywords, Boolean operators, synonyms, and truncation was employed to broaden the search results. The reviewer pre-tested the search strategy on PubMed/MEDLINE in consultation with a trained librarian (Supplementary Material Table S2). Once the PubMed search strategy was finalized, it was adapted for other databases such as Web of Science, EMBASE, and Scopus. Google Scholar was also searched for up to 10 pages. The MesH terminologies used were (developmental disability * or intellectual disability * or special need * or intellectual disabilities or disabled * or autism * or ADHD or ASD, cerebral palsy or attention deficit hyperactivity disorder or Down syndrome or Fragile X Syndrome or fetal alcohol spectrum disorder) AND ((oral or dental) adj (health, intervention, treatment, procedure, hygiene, or anxiety)) stress, psychological/adaptation, psychological/or psychological distress/or dental anxiety/or sensation/or patient compliance/or “treatment adherence and compliance”/or oral health/or oral hygiene. A manual search for reference lists from relevant articles was conducted to match the inclusion criteria, and previously published scoping reviews were reviewed to identify eligible studies.

The electronic databases were searched with a language restriction and a publication date range from January 2007 to August 2024. The final search was conducted on 14 September 2024 and was updated on 16 November 2024.

### 2.3. Selection Process

**Figure 1:**
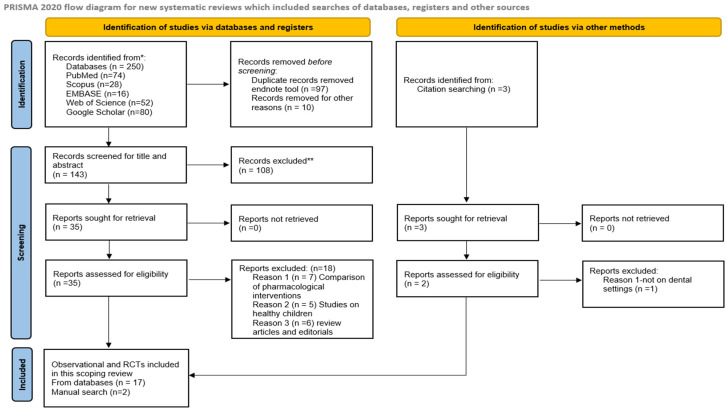
PRISMA-ScR for inclusion of studies. * Consider, if feasible to do so, reporting the number of records identified from each database or register searched (rather than the total number across all databases/registers); ** If automation tools were used, indicate how many records were excluded by a human and how many were excluded by automation tools. Source: Page M.J. et al. BMJ 2021; 372:n71. doi:10.1136/bmj.n71 visit https://creativecommons.org/licenses/by/4.0/ (accessed on 18 December 2024).

Studies identified through various databases and hand searching were imported into EndNote 20, and duplicates were removed. Following a pilot test, the reviewer carefully screened the titles and abstracts according to strict inclusion criteria. If the abstract was unclear, the full text was retrieved to determine eligibility for inclusion. Articles that met the inclusion criteria were retrieved and reviewed in detail. The author contacted a pediatric surgeon to seek additional information or verify findings from the included articles, reducing the likelihood of missing any critical data. A total of two revisions were conducted to ensure the accurate extraction of findings from the included articles. The included studies were analyzed, and those not meeting the inclusion criteria were excluded (Supplementary Table S3). The selection process was summarized using the PRISMA-ScR (Preferred Reporting Items for Systematic Reviews and Meta-Analyses for Scoping Reviews) framework [13] (Figure 1).

### 2.4. Data Collection

After developing a standardized data extraction form, it was piloted in two studies and subsequently refined to ensure all relevant data were captured. The reviewer extracted the data, which were then sent to a pediatric dentist (MA) for verification to ensure accuracy. If any information was missing, the authors of the included articles were contacted. Three contact attempts were made, and if there was no response, the data were extracted based on the information available. The extracted data were entered into a Microsoft Excel sheet, including specific details such as author, country, year, study design, participant information, non-pharmacological intervention details and descriptions, outcome measures, and conclusions.

## 3. Results

A total of 250 studies were extracted from various databases, of which 107 duplicate data were removed. Of the remaining 143 studies, a thorough evaluation of the title and abstract was performed, 108 studies were removed, and 35 articles were finally included for full-text reading. Of these, 18 studies were excluded for various reasons, such as using pharmacological interventions, being review articles, and the lack of a measurement of anxiety levels. Finally, a total of 17 studies were included in database searching.

A manual search for references from included articles was performed, and two articles were retrieved for inclusion. Following this, 19 studies evaluating dental anxiety management with non-pharmacological intervention for children with special needs were included in this review (Figure 1).

### 3.1. Description of Included Studies

Nineteen studies, including fifteen randomized controlled trials [8,15,16,17,18,19,20,21,22,23,24,25,26] and four prospective cohort studies [27,28,29,30], were included in the current scoping review. All the research included has recorded the effectiveness of various non-pharmacological interventions like visual pedology, audio–visual distraction, visual distraction, SADE, animations, tell–show–do, and picture exchange communication systems for reducing dental anxiety among SNC patients undergoing various dental treatments. The studies were conducted in multiple countries like the USA, India, Iran, Israel, Italy, Saudi Arabia, and UAE and included SNC patients aged 3–19 years. Among the included studies, some were on patients with no dental experience and patients with severe dental anxiety (Table 2).

**Figure 2:**
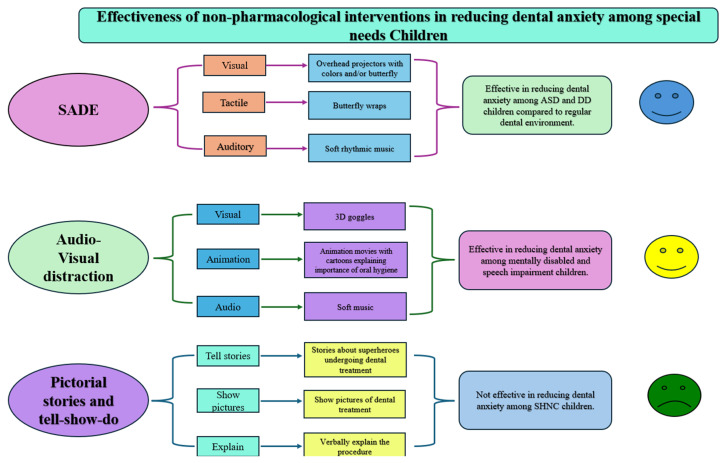
Effectiveness of non-pharmacological interventions on reduction in dental anxiety.

The effectiveness of the non-pharmacological intervention was measured in all the included studies, especially in reducing dental anxiety, the number of appointments required to complete the treatment, and the physician rating scale. The non-pharmacological interventions utilized in the studies were used to facilitate basic and noninvasive dental treatments like dental examination, restorations, scaling, oral prophylaxis, and fluoride applications. Figure 2 illustrates a conceptual understanding of the effectiveness of non-pharmacological interventions in reducing dental anxiety among SNC patients.

### 3.2. Sensory Adaptive Dental Environnent (SADE)

Seven randomized controlled trials in this review evaluated the effect of a Sensory Assisted Dental Environment (SADE) on reducing dental anxiety among special needs patients [16,19,22,23,24,25,31]. The studies involved children aged 3–12 years with developmental disorders, mental disabilities, or autism spectrum disorder. These trials were conducted in various countries, including the USA, Israel, and Italy. One randomized controlled trial in Israel specifically investigated the effect of a Snoezelen SADE on reducing dental anxiety in patients undergoing dental prophylaxis. In a Snoezelen dental environment, the clinic is designed with low or special lighting effects, soft and relaxing music, vibrations from the dental chair, and the application of deep pressure using an X-ray vest [19]. This approach is primarily client-centric, aiming to alleviate dental anxiety in special needs patients.

To measure dental anxiety, the included RCTs employed specific tools such as negative dental behavior checklists, electrodermal activity monitoring, the Child and Adolescent Inventory Anxiety Scale, Venham’s Picture Test, and the Frankl Scale. All the included studies aimed to compare patient behavior during dental treatment in SADE versus regular dental (RD) environments.

The studies included showed mixed results. For instance, one study reported a significant improvement in children’s behavior as recorded by the dental hygienist, while another study noted only a slight improvement based on the Children’s Dental Behavior Rating Scale. The study by Stein et al. (2014) found minimal to no improvement in children’s behavior when using SADE techniques to reduce dental anxiety [25]. A study by Fakhruddin et al., 2019, on patients with hearing impairment reported that SADE intervention along with virtual reality was helpful in managing patients [8]. Most of the included studies tried SADE with other behavior intervention to check the effectiveness of non-pharmacological interventions on special need children. However, despite these variations, all the included studies favored SADE as an effective approach for reducing dental anxiety among special needs children (SNCs).

### 3.3. Virtual Reality (VR)

Two randomized controlled trials and three cross-sectional studies conducted in the USA, India, and Saudi Arabia evaluated virtual reality (VR) techniques for patients with special needs. Fakruddin et al. (2019) studied the anxious behavior of children with hearing impairments undergoing pulpectomy treatment [8]. The authors followed two techniques: the tell–show–do method and virtual reality. In the first appointment, the procedure was explained to the children using the tell–show–do method, and their anxiety levels were recorded using a smiley face scale [8]. During the second appointment, virtual reality techniques, including 3D glasses, were employed, and anxiety levels were assessed again. The study found no significant difference in the patient’s anxiety levels between the two techniques. However, the authors suggested that combining the tell–show–do method with virtual reality could help reduce dental anxiety [8].

In the cross-sectional study conducted by Kheraif et al., artificial intelligence (AI) and virtual reality (VR) were utilized to reduce dental anxiety among patients with mental illness [28]. The study measured dental anxiety using the Venham Anxiety Scale and a galvanic skin response sensor. The authors reported that the use of VR and AI in a dental clinical setting significantly reduced anxiety levels in children with mental disabilities [28]. In another study by Kheraif et al., autistic children experienced a reduction in dental anxiety when exposed to VR during basic dental procedures [26]. Kaur et al., in their randomized controlled trial, studied the anxiety levels of children with hearing and speech impairments [18]. The authors randomly divided the children into three groups: Group A received no intervention, Group B used visual distraction through VR glasses during dental treatment, and Group C was distracted with videos of their favorite cartoons. The study results showed a significant reduction in anxiety levels, measured using a pulse oximeter, for children in Group B compared to the other groups [18]. Overall, all the reviewed studies reported decreased dental anxiety when VR was utilized during dental treatment for special needs patients.

### 3.4. Audio–Visual Distraction (AVD)

Three randomized controlled trials [15,25,32] and one cross-sectional study [18] conducted in the United States, India, and Italy evaluated audio–visual distraction (AVD) techniques to reduce dental anxiety among special needs patients. In one trial involving children with autism spectrum disorder, video goggles, modeling, and music were used to reduce anxiety and improve behavior [31]. Like virtual distraction, the audio–visual distraction intervention in this study had significantly positive outcomes in reducing dental anxiety.

The other two randomized trials investigated the use of AVD for dental restorations among SNC patients. One study included children with Down syndrome and used the Frankl Scale to measure anxiety levels [15]. The findings indicated that AVD effectively reduced dental anxiety in the second session compared to the first. The second study evaluated children with mild intellectual disabilities using Venham’s Anxiety Rating Scale to assess reductions in anxiety during restorative treatment [32]. Anxiety levels were measured at two intervals: during the initial visit and one month later during dental treatment. The results showed a significant decrease in dental anxiety during the second interval [15]. Overall, all the included studies favored the use of AVD in the children with special needs.

### 3.5. Visual Pedagogy

A randomized controlled trial (RCT) conducted in Iran evaluated the effect of visual pedagogy on reducing anxiety levels in children with autism spectrum disorder [20]. The participants were aged between 6 and 12 years. The authors used coloring pictures to illustrate dental procedures to the children at each visit. The study assessed the children’s behavior using the Frankl Behavior Rating Scale to evaluate the responses of both groups [20]. The results showed no significant difference in anxiety levels among participants after using visual pedagogy. The study concluded that visual pedagogy has minimal or no effect on reducing dental anxiety in autistic children [20].

### 3.6. Music Distraction

A crossover study by Gowdham et al. in India evaluated the effect of music on reducing dental anxiety in children with mild intellectual disabilities [29]. The study included children aged 3–12 years, and trained dentists measured anxiety levels using the galvanic skin response. The special needs patients (SNCs) were randomly divided into two groups: Group A received no intervention, while slow music was played for Group B. The study reported no significant difference in anxiety levels between the groups [29]. However, a mild reduction in anxiety was observed while the music was playing.

**Figure 3:**
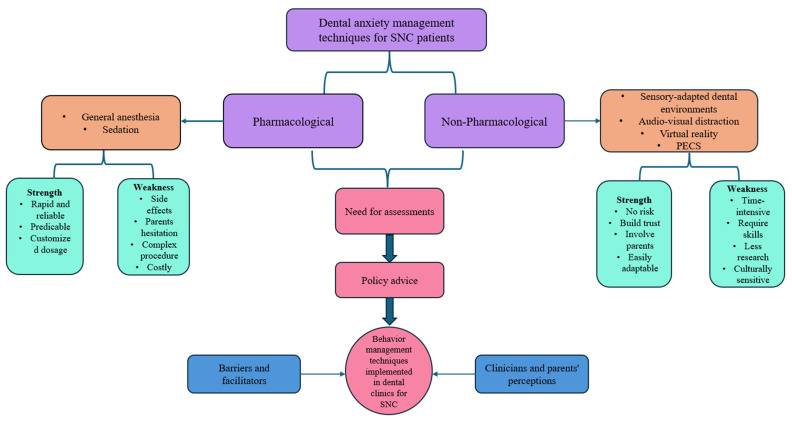
Conceptual map for dentists and policymakers to evaluate the need for dental anxiety management for SNC patients.

Even though the included studies have demonstrated positive outcomes with the implementation of non-pharmacological techniques to reduce dental anxiety, it is crucial to understand the necessity of these interventions. Limited research focuses on special needs children (SNCs), unique oral healthcare requirements, and behavior modification needs. Furthermore, understanding the perceptions of dentists, parents, and caregivers regarding oral healthcare for this vulnerable population is essential. The conceptual map developed in this study highlights the strengths and weaknesses of pharmacological and non-pharmacological behavioral interventions for SNCs. These insights should be considered by policymakers, pediatric dentists, and general practitioners when designing interventions to reduce dental anxiety in this patient group (Figure 3).

## 4. Discussion

The findings of this scoping review show that oral health needs assessment and requirements for pharmacological and non-pharmacological interventions for behavior management should be considered as starting points before planning any oral health treatment for SNC patients. This need assessment should cover all the areas, including invasive and noninvasive dental treatment and pharmacological and non-pharmacological behavior modification techniques.

### 4.1. Summary of the Main Results

This scoping review consists of 15 randomized controlled trials [8,15,16,17,18,19,20,21,22,23,24,25,26,31,32] and four observational studies [27,28,29,30] investigating the effectiveness of the non-pharmacological intervention on the behavior modification of SNC patients. The included studies used non-pharmacological interventions, SADE, virtual reality, artificial intelligence, and audio–visual distraction to reduce dental anxiety in dental clinical settings and enhance cooperative behavior during invasive and noninvasive dental treatment. Results from the included studies on tell–show–do and video modeling were inconclusive. The included studies on virtual reality audio–visual distraction showed trivial to large effects on anxiety and pain reduction, mainly when the 3D eyeglasses were worn during restorative and pulp canal treatment [27,28]. However, results from pictural presentation and music distraction were inconclusive [20,29].

Seven RCTs examined the effectiveness of SADE and found trivial to moderate effects on the reduction in anxiety and behavior modification [8,16,19,22,23,24,25]. Two previously published systematic reviews performed on autistic children and mentally disabled children found a positive impact of the SADE on behavior modification [2,5]. Considering the significant reduction in dental anxiety and cost-effective implementation, SADE represents a feasible behavior modification approach to control anxious SNC patients [3]. The study included a pedagogical method (colorful pictures) and found that implementing mobile applications via pictorial presentation can improve cognitive behavior [20]. However, the application used in the study is commercially unavailable; other applications utilized for maintaining the oral hygiene of autistic children are commercially available. While this and other mobile applications have not been researched independently, very low-quality evidence supports pictorial presentation in behavior management.

Of the studies in this scoping review, each focused on a single behavior modification technique, seven on SADE [16,19,22,23,24,25,31], four on virtual reality [18,27,28,30], three on AVD [15,21,32], and one on positive imaginary [17] and video modeling [20]. The chronological age of children in the studies varied from 3 years to 19 years, respectively. The samples selected in the included studies also varied, with some involving autistic children, others with children diagnosed with mental and developmental illness, and others with children suffering from hearing and speech loss. The included studies exhibited significant variation in the distress- and cooperation-related outcome measures and in the quality of their assessments. This heterogeneity highlights the varied effectiveness and uncertain reliability of evidence concerning the behavior guidance techniques studied specifically among SNC patients. Despite these limitations, the lack of systematic and scoping reviews focusing on this population and the inability to compare similar outcomes emphasize the importance of these findings. Techniques such as positive imagery, audio–visual distraction (AV), SADE, and virtual reality emerge as promising approaches for behavior guidance in SNC patients.

### 4.2. Non-Pharmacological Interventions in Reducing Dental Anxiety

In the literature, there are few studies focusing on dental anxiety management among children with special needs [8,9,18,21]. Most published studies have focused on SADE along with pharmacological management, like giving general anesthesia for managing dental anxiety for invasive treatments among SNCs [16,31]. A network meta-analysis on the management of dental anxiety among pediatric patients reported that musical intervention can be helpful in reducing dental anxiety among children with a mild intellectual disability [33].

In a systematic review on managing children with autistic disorder, virtual reality was mentioned as one of the best approaches [34]. However, in children with mental disabilities, VR was not appropriate. Children with an intellectual disability experience cognitive impairment and communication difficulties, which lead dentists to perform pharmacological interventions for managing these children. Non-pharmacological interventions like musical distraction, AVD, SADE, and VR, which are cost-effective, and a noninvasive approach can be helpful in managing dental anxiety among children with special needs.

With the advancement and rapid development of non-pharmacological intervention among SNCs, certain interventions are helpful in children with mild to moderate dental anxiety. However, the applicability of these interventions for highly anxious SNCs needs to be verified. Therefore, it is not possible to draw definitive conclusions regarding recommended non-pharmacological interventions for children with special needs with varying degrees of dental anxiety. Further analysis and validation are required to draw a conclusive remark on the management of children with special needs.

Glassman et al. reviewed guidelines about pharmacological interventions like GA and conscious sedation and concluded that there is lack of protocol targeting pediatric patients with special needs [35]. Moreover, the authors reported that there were only a few publications focusing on dental anxiety management for children with special needs. Hence, there is an urgent need for the researchers to focus on developing guidelines for managing these vulnerable populations.

### 4.3. Comparison of Non-Pharmacological Interventions

Most of the included studies focused on a single non-pharmacological intervention for managing dental anxiety among children with special needs (SNCs). Among these, SADE was identified as the most effective management technique. Distraction techniques, which are safe and cost-effective, enhance the overall experience for SNC patients undergoing invasive and painful dental procedures by diverting their attention from unpleasant procedures. However, the included studies reported inconclusive results, which could be attributed to the type of non-pharmacological intervention used for different special needs. For instance, studies on mentally disabled children commonly employed SADE, while autistic children primarily benefited from audiovisual distraction (AVD) and virtual distraction. For hearing-impaired children, a combination of the tell–show–do method and virtual distraction was used. These behavioral modification techniques were mostly applied to noninvasive procedures. Fakruddin et al., in their study on endodontic treatment for hearing-impaired children, reported no changes in anxiety levels. This highlights the importance of considering the specific treatment needs of children. Notably, all the included studies focused on noninvasive dental treatments and supported the use of non-pharmacological interventions.

Inconsistencies were observed in the effectiveness of visual pedology and modeling. This could be due to the possibilities of age group and special need involved. Nilchin et al., in their study of autistic children, reported no change in the anxiety levels of children, even for basic dental diagnostic procedures [20]. One possible explanation for this could be the reduced cognitive function of children with ASD, which could impact their behavior during dental treatment. In the treatment of SNC patients with poor dental health and dental anxiety, recognizing the clinical characteristics of patients helps dentists to make appropriate decisions on behavior modification techniques and provide high-quality dental care.

### 4.4. Strengths and Limitations

The current scoping review is notable for its methodological rigor, as it adhered to all the essential steps required for such studies. This review aimed to address a clinical question concerning the management of anxiety in children with special needs, a group that has been understudied. Notably, this is the first scoping review to focus specifically on managing anxiety in special needs children using non-pharmacological interventions. Additionally, the conceptual map created in this scoping review can provide insights for policymakers and pediatric and general practitioners to design interventions that can help SNC patients.

Only a limited number of studies have evaluated the effectiveness of non-pharmacological interventions among special needs children (SNC) patients. Due to this limitation, the author combined invasive and noninvasive dental procedures in this scoping review. The American Academy of Pediatric Dentistry has recommended using VR, video modeling, and audio–visual distraction in combination with tell–show–do, positive reinforcement, and relaxation techniques to reduce dental anxiety. These behavior modification techniques have primarily been studied in healthy children. However, there is a scarcity of articles focusing on behavior modification and anxiety reduction in children with special needs. A conclusive remark cannot be provided in this review due to the limitation in published articles, different SNC populations considered, and different interventions used.

The SADE (Sensory Adapted Dental Environment) was specifically developed for SNC patients, but its utilization in dental settings remains minimal. Moreover, most included studies lacked control groups, making it impossible to compare the effectiveness of two or more behavior modification techniques in SNC patients. A similar issue arose with the instruments used to measure anxiety levels in SNC patients, preventing this review from determining which instrument is superior.

### 4.5. Future Recommendations

Only nineteen studies have focused on reducing dental anxiety in special needs children (SNCs) during dental treatment, emphasizing the need for more clinical trials and cross-sectional studies to evaluate the effectiveness of behavior modification techniques in this population. Additional research is required to assess the proposed behavior guidance approaches for dental professionals working with SNC patients. Future studies should also explore whether modifications to these methods enhance their efficacy for children and youth with special healthcare needs.

Investigating the standardized or manualized implementation of these techniques would be valuable. Moreover, subsequent research should examine current clinical practices in applying behavior guidance techniques, particularly in SNC patients. Disseminating such studies would provide more relevant and substantial data for clinicians to adopt appropriate techniques for managing dental anxiety in this population.

It is recommended that high-quality prospective studies focus on various behavioral guidance techniques and, where feasible, implement randomized controlled trials (RCTs). These RCTs should adhere to the Consolidated Standards of Reporting Trials (CONSORT) reporting guidelines to ensure methodological rigor and reliability.

## 5. Conclusions

The present scoping review identified three techniques: virtual reality (VR), audio–visual distraction (AVD), and the Sensory Adapted Dental Environment (SADE) as effective tools for reducing dental anxiety among special needs children (SNCs). In most of the studies reviewed, SADE tools combined with visual distraction effectively managed dental anxiety and promoted positive behavior in patients. Even studies focusing on traditional behavior management methods suggest that combining conventional and non-pharmacological interventions could be beneficial in managing SNC patients. Furthermore, combining these techniques could assist dentists in building a bond with SNC patients and their caregivers, fostering a positive approach to patient education and treatment. Tools such as VR, audio–visual goggles and visors, augmented reality (AR), and artificial intelligence (AI) show significant potential for improving behavior management and reducing dental anxiety and treatment avoidance among SNC patients. Future studies should focus extensively on the potential of these technologies to reduce dental anxiety and enhance behavior management in SNC patients. This approach will help dentists and caregivers ensure proper treatment for this vulnerable population.

## Figures and Tables

**Table 1:** Inclusion and exclusion criteria.

Inclusion	Exclusion
Studies that included children with special needs between 3 and 19 years of age.	Studies that included healthy children and/or SNCs 19 years or older.
Studies on non-pharmacological management of dental anxiety among SNC group.	Studies on pharmacological interventions.
Observational studies and randomized control trials.	Reviews, conference proceedings, editorials, and the gray literature.
All types of non-pharmacological interventions are used to manage dental fear among SNCs.	Any other intervention or mixed interventions for managing dental anxiety among healthy children.
Studies published in English beginning from 2007.	Studies published in any other language or in English but published before 2007.

**Table 2:** Characteristics of included studies.

Sr. No.	Author/Year/Country	Study Design	Sample Size/Age	Characteristics of Participants	Anxiety Measuring Scales	Dental Treatment Received	Non-Pharmacological Intervention	Statistical Findings	Outcomes
1.	Shapiro et al. 2007, Israel [23]	RCT	SS: 19 AG: 6–9	Developmental disability	Dermal resistance and video recorder	Dental scaling	Sensory-adapted dental environment (SDE)	No significant difference in anxious behavior was found in the RDE and SDE environments.	SDE demonstrated an important aspect in decreasing anxiety levels.
2.	Shapiro et al. 2008, Israel [22]	RCT	SS: 16 AG: 6–11	Developmental disability	Electrodermal activity (EDA)	Dental diagnosis	SADE	The mean duration of anxious behaviors was significantly reduced in the SADE compared to the RDE, *p* < 0.01.	Noninvasive environmental approach helps reduce dental anxiety among DD children.
3.	Shapiro et al. 2009, Israel [24]	RCT	SS: 16 AG: 6–11	Developmental disability	Changes in palmar skin conductance by means of electrodes	Any dental treatment	Sensory-adapted environment (SAE)	The statistically significant mean duration of anxious behaviors in the SAE and RE for DD children (*p* < 0.001).	A sensory-controlled environment may represent an important substitute for the commonly used alternatives of pharmacological sedation.
4.	Aminabadi et al. 2011, Iran [26]	RCT	SS: 8 0AG: 6–7	Mildly mentally disabled	Modified child dental anxiety scale	Noninvasive dental treatment	Pictorial story	Statistically significant lower situational anxiety among the test group (*p* < 0.001, F = 271.024).	Pictorial stories can help reduce dental anxiety among children with intellectual disabilities.
5.	Stein et al. 2014, USA [25]	RCT	SS: 44 AG: 6–12	ASD	Child and adolescent symptom inventory anxiety scale (CASI-Anx)	Regular dental treatment	SADE	Statistical significance was reported among the anxiety levels and age of patients with ASD (*p* = 0.001).	Even with the SADE environment in dental clinics, ASD children experience more dental anxiety compared to healthy children.
6.	Isong et al. 2014, USA [17]	RCT	SS: 8 0AG: 7–19	ASD	Venham anxiety scale	Dental diagnosis	Video peer modeling and video Goggles	No statistical difference was recorded among the groups.	The result of this pilot study suggests that electronic media might be useful in reducing dental anxiety among ASD children.
7.	Cermak et al. 2015, USA [16]	RCT	SS: 44 AG: 6–17	ASD	Venham’s picture test and child and adolescent symptom inventory anxiety scale	Dental treatment	SADE	The anxiety and cooperation scale showed an effect size of 0.13 in ASD children.	SADE is recommended for reducing dental anxiety among children with ASD and other disabilities.
8.	Nilchian et al. 2017, Iran [20]	RCT	SS: 4 0AG: 6–12	ASD	Frankl scale	Flouride application	Pedagogical method (colorful pictures)	No significant difference in anxiety scores was analyzed among the test and control groups.	Visual pedagogy is not effective in reducing anxiety and improving ASD children’s behavior in dental clinics.
9.	Fakhruddin et al. 2019, United Arab Emirates [8]	RCT	SS: 15 AG: 5–7	Hearing-disabled children	Smiley Faces Program (SFP)	Endodontic treatment	Tell–show–do and visual distraction	No statistical significance was recorded. However, the anxiety score decreased in the patients after the behavioral intervention.	A combination of tell–show–do and visual distraction works better for children with hearing impairment.
10.	Kim et al. 2019, USA [19]	RCT	SS: 21 AG: 6–19	Developmental disabilities	Frankl scale	Dental diagnostic	Sensory-adapted dental environment (SADE)	The median difference in Frankl scores was 1, favoring better behavior in SADE.	SADE improves DD children’s perception toward dental treatment.
11.	Rao et al. 2019, India [22]	RCT	SS: 3 0AG: 6–10	Developmental anomalies	Physiologically, by measuring pulse rate and oxygen saturation levels using pulse oximeter	Restorative treatment	Virtual reality distraction	Very high statistical significance in the reduction in pain perception and anxiety levels (*p* < 0.0001).	Virtual reality distraction helps reduce anxiety among SNCs.
12.	Suresh and George 2019, India [31]	Cross-sectional study	SS: 68 AG: 8–15	ASD	Venham’s picture test	Noninvasive dental treatment	Virtual reality	Statistical reduction in anxiety score (*p*-value = 0.017).	Virtual reality distraction can be used as a successful behavior management method in autistic children during routine dental treatment.
13.	Bagattoni et.al 2020, Italy [15]	RCT	SS: 48 AG: 5–12	Down syndrome	Frankl scale	Restorative treatment	Audio–visual distraction	The difference between the groups was significant (*p* = 0.015).	The results of this study showed that audio–visual measures cannot replace conventional treatment for reducing anxiety among children suffering from DS.
14.	Kaur et al. 2021, India [18]	RCT	SS: 24 AG: 6–14	Hearing and speaking disability	Pictorial scale (PJS)	Primary dental treatment	Virtual reality	A significant difference was observed among children’s anxiety levels before and after treatment (*p*-value = 0.005).	Video distraction using VR glasses is the perfect measure to reduce dental anxiety.
15.	Gowdham et al. 2021, India [29]	Crossover design	SS: 2 0AG: 4–14	Children with mild to severe intellectual disabilities	Galvanic skin response (GSR)	Routine dental examination	Music therapy	In both the dental appointments, the GSR value was statistically significant (*p*-value = 0.0002).	Musical distraction has a positive impact on reducing dental anxiety in intellectually disabled children.
16.	Fallea et al. 2022, Italy [31]	RCT	SS: 5 0AG: 4–10	Mentally disabled children	Not mentioned	Routine dental examination	Sensory-adapted dental environment (SADE)	Statistical significance for male patients; chi-squared test = 18.225, df = 1, *p*-value = 0.00002.	SADE has a positive impact on treatment outcomes among male patients.
17.	Kheraif et al. 2023, Saudi Arabia [28]	Cross-sectional	SS: 9 0AG: 6	Mental disability	Galvanic skin response sensor and Venham anxiety scale	Any dental treatment	Virtual reality and artificial intelligence	Graphs were made to represent the anxiety scores, and after utilizing VR and AI, the graphs showed a significant decrease in anxiety.	A significant decrease in anxiety was observed in children with mental disabilities during routine, noninvasive dental treatment procedures using VR-based distraction.
18.	Mehrotra et al. 2023, India [32]	RCT	SS: 2 0AG: 7–12	Mild intellectual disability	Venham’s anxiety rating scale	Restoration and dental diagnosis	Audio–visual distraction aids	The anxiety scale was significantly associated with visual distraction aids (<0.05).	This study highly recommends the utilization of audio–visual techniques to reduce dental anxiety among intellectually disabled children.
19.	Kheraif et al. 2024, Saudi Arabia [27]	Cross-sectional	SS: 14 0AG: 4–16	ASD	Venham anxiety and behavior scale (VABS)	Dental examination	Virtual reality	VR significantly reduces anxiety levels among ASD children (*p*-value < 0.05).	Integrated VR technology improves the dental experience of children with ASD.

SS: sample size; AG: Age group; ASD: Autism spectrum disorder; SADE: sensory-adapted dental environment; RDE/RE: regular dental environment.

## Data Availability

Can be provided on request from the corresponding author.

## Supplementary Materials

The following supporting information can be downloaded at: https://www.mdpi.com/article/10.3390/children12020165/s1. Supplementary Materials contain PRISMA-ScR checklists, search terminologies, and excluded articles.

## Abbreviations

The following abbreviations are used in this manuscript:

SN	Special needs
PCC	Population, context, and content
SADE	Sensory adaptive dental environment
AVD	Audio–visual distraction
VD	Visual distraction
DA	Dental anxiety
SNHC	Special need healthcare children
