# Supplementary material for: Effectiveness of Non-Pharmacological Interventions in Reducing Dental Anxiety Among Children with Special Needs: A Scoping Review with Conceptual Map
Source: Children (Basel). 2025 Jan 29;12(2):165. doi: 10.3390/children12020165 (PMC11854481; doi:10.3390/children12020165)
Supplement: Supplementary file 1 [file children-12-00165-s001.zip › Supplementary table S2 SNC.pdf]

Supplementary table 2: Various terminologies used, and articles found on electronic databases

Electronic databases	Terminologies	Article found
PubMed	1. (Child* or adolescent* or teen or pediatric* or preschool or infant*).ti,ab.<br>2. Child, Preschool/ or Pediatrics/ or Adolescent/ or Child/ or infant/ or young adult/<br>3. 1 or 2<br>4. (Developmental disability* or intellectual disability* or special need* or intellectual disabilities or disable* or autism* or ADHD or ASD or Cerebral palsy or attention deficit hyperactivity disorder or Down Syndrome or Fragile X Syndrome or Fetal alcohol spectrum disorder).ti,ab.<br>5. Attention Deficit Disorder with Hyperactivity/ or Cerebral Palsy/ or Autistic Disorder/ or Disabled Persons/ or Disabled Children/ or child development disorders, pervasive/ or developmental disabilities/ or intellectual disability/ or Down Syndrome/ or Fragile X Syndrome/ or Fetal Alcohol Spectrum Disorders/<br>6. 4 or 5<br>7. 3 and 6<br>8. ((Dental adj3 (sensory adapted environment* or multi-sensory environment*)) or Snoezelen or SADE).ti,ab.<br>9. Health facility environment/ or environment/ or dental offices/ or environmental adaption/ or Environment, Controlled/<br>10. 8 or 9<br>11. (((Oral or dental) adj (health, intervention, treatment, procedure, hygiene, or anxiety)) ti,ab.<br>12. Stress, Psychological/ or Adaptation, Psychological/ or Psychological Distress/ or Dental Anxiety/ or Sensation/ or Patient Compliance/ or "Treatment Adherence and Compliance"/ or Oral Health/ or Oral hygiene/	74

	13. 11 or 12<br>14. 7 and 10 and 13	
Web of science	1. Child* or adolescent* or teen or youth or young adult or pediatric* or preschool or infant* (Title) or Child* or adolescent* or teen or youth or young adult or pe?diatric* or preschool or infant* (Abstract)<br>2. Developmental disability* or intellectual disability* or special need* or mental retardation or disable* or autism* or ADHD or ASD or Cerebral palsy or attention deficit hyperactivity disorder or Down Syndrome or Fragile X Syndrome or Fetal alcohol spectrum disorder (Title) or Developmental disability* or intellectual disability* or special need* or mental retardation or disable* or autism* or ADHD or ASD or Cerebral palsy or attention deficit hyperactivity disorder or Down Syndrome or Fragile X Syndrome or Fetal alcohol spectrum disorder (Abstract)<br>3. TI=(Dental "NEAR/3" (sensory adapted environment* or multi-sensory environment*) or Snoezelen or SADE or SAE) or Snoezelen or SADE or SAE)<br>4. ((Oral or dental) "NEAR/1" (health, or hygiene, or anxiety)) or behavior or compliance or physiological or pain or arousal or stress or psychological (Title) or (Oral or dental) "NEAR/1" (health or hygiene or anxiety)) or behavior or compliance or physiological or pain or arousal or stress or psychological (Abstract)<br>5. #1 AND #2<br>6. #3 AND #4 AND #5	52
Embase	1. (Child* or adolescent* or teen or youth or young adult or pediatric* or preschool or infant*).ti,ab.<br>2. preschool child/ or pediatrics/ or adolescent/ or child/ or preschool child/ or school child/ or infant/ or young adult/<br>3. 1 or 2<br>4. (Developmental disability* or intellectual disability* or special need* or intellectual disabilities or disable* or autism* or ADHD or ASD or	16

	Cerebral palsy or attention deficit hyperactivity disorder or Down Syndrome or Fragile X Syndrome or Fetal alcohol spectrum disorder).ti,ab.<br>5. Disabled Persons/ or autism/ or child with disabilities/ or developmental disorder/ or intellectual impairment/ or cerebral palsy/ or attention deficit disorder/ or Down syndrome/ or fragile X syndrome/ or fetal alcohol syndrome/<br>6. 4 or 5<br>7. 3 and 6<br>8. ((Dental adj3 (sensory adapted environment* or Multi-sensory environment*)) or Snoezelen or SADE).ti,ab.<br>9. health care facility/ or environment/ or dental facility/<br>10. 8 or 9<br>11. (((Oral or dental) adj (health or hygiene or anxiety)) or behavior or compliance or physiological or pain or arousal or stress or psychological).ti,ab.<br>12. mouth hygiene/ or patient compliance/ or sensation/ or dental anxiety/ or pain/ or nociception/ or arousal/ or psychological adjustment/ or mental stress/<br>13. 11 or 12	
Scopus	(TITLE-ABS-KEY("dental treatment" OR "dentistry" OR "dental care") AND TITLE-ABS-KEY("dental anxiety" OR "dental fear" OR "dental fears" OR "Odontophobia" OR "Odontophobias" OR "Dental Anxieties" OR "behavior" OR "behaviors" OR "pain" OR "dental pain" OR "behavior management" OR "behavior guidance" OR "Behavior Modification" OR "Behavior modifications")) AND ("child" OR "preschool children" OR "adolescent") AND (LIMIT-TO(DOCTYPE,"ar")) AND (LIMIT-TO(SUBJAREA,"DENT") AND ("clinical trial" OR "randomized controlled trial" "controlled clinical trial"))	28
Google Scholar	Child, Preschool/ or Pediatrics/ or Adolescent/ or Child/ or infant/ or young adult/(Developmental disability* or intellectual disability* or special need* or mental retardation or disable* or autism* or ADHD or ASD or	80

	Cerebral palsy or attention deficit hyperactivity disorder or Down Syndrome or Fragile X Syndrome or Fetal alcohol spectrum disorder). (((Oral or dental) adj (health, intervention, treatment, procedure, hygiene, or anxiety)) Stress, Psychological/ Adaptation, Psychological/ or Psychological Distress/ or Dental Anxiety/ or Sensation/ or Patient Compliance/ or "Treatment Adherence and Compliance"/ or Oral Health/ or Oral hygiene	
Total		250
