# Supplementary material for: Effectiveness of Non-Pharmacological Interventions in Reducing Dental Anxiety Among Children with Special Needs: A Scoping Review with Conceptual Map
Source: Children (Basel). 2025 Jan 29;12(2):165. doi: 10.3390/children12020165 (PMC11854481; doi:10.3390/children12020165)
Supplement: Supplementary file 1 [file children-12-00165-s001.zip › Supplementary table S3.pdf]

Supplementary Table S3: Reason for exclusion

**Author/s and Year Published**	**Reason/s for Exclusion**
Aziz (2022)	Not on special need children
Unwin, Powell & Jones. (2022)	Not in a dental setting.
Shapiro et al. (1997)	Not in year of publication
Kopel (1977)	Not a clinical trial.
Novakovic et al. (2019)	Exceeded inclusion criteria age group.
Mitchell & Gaskin. (2004)	Not a clinical trial.
Zainab Juma Jafar	Feasibility study. Inappropriate outcome measure.
Aljubour et al. (2022)	Not on special need children
Fava & Strauss. (2010)	Not in dental setting.
Peivand Bastani (2022)	Not on dental anxiety
Vineet Dhar (2022)	Systematic review
Potter, Wetzel & Learman. (2018)	Exceeded inclusion criteria age group.
Lahman et al. (2008)	Not IDD population.
Ghadimi et al. (2018)	Not IDD population
Hamdan Alamri (2022)	Review article
Chen & Hawkins. (2021).	Pre/post design. No randomisation

Soares Salles, (2011)	Age group more than 21
Cajares, Rutledge & Haney (2016)	Involved sedation during treatment.
Chen et al. (2012)	Not RCT, no control.
